# Characterization of Endocannabinoid System and Interleukin Profiles in Ovine AEC: Cannabinoid Receptors Type-1 and Type-2 as Key Effectors of Pro-Inflammatory Response

**DOI:** 10.3390/cells9041008

**Published:** 2020-04-18

**Authors:** Luana Greco, Valentina Russo, Cinzia Rapino, Clara Di Germanio, Filomena Fezza, Nicola Bernabò, Paolo Berardinelli, Alessia Peserico, Domenico Fazio, Mauro Maccarrone, Mauro Mattioli, Barbara Barboni

**Affiliations:** 1 Faculty of Bioscience and Agri-Food and Environmental Technology, University of Teramo, 64100 Teramo, Italy; lgreco@unite.it (L.G.); nbernabo@unite.it (N.B.); pberardinelli@unite.it (P.B.); apeserico@unite.it (A.P.); mmattioli@unite.it (M.M.); bbarboni@unite.it (B.B.); 2 Faculty of Veterinary Medicine, University of Teramo, 64100 Teramo, Italy; crapino@unite.it; 3 Immunology Core, Vitalant Research Institute, San Francisco, CA 94118-4417, USA; cdigermanio@vitalant.org; 4 Department of Experimental Medicine, Tor Vergata University of Rome, Via Montpellier 1, 00121 Rome, Italy; filomena.fezza@uniroma2.it; 5 European Center for Brain Research/IRCCS S. Lucia Foundation, 00179 Rome, Italy; dfazio@unite.it (D.F.); mmaccarrone@unicampus.it (M.M.); 6 Department of Medicine, Campus Bio-Medico University of Rome, 00128 Rome, Italy

**Keywords:** amniotic epithelial cells, cannabinoid receptors, immunomodulation

## Abstract

Amniotic epithelial cells (AEC) have been proposed as promising clinical candidates for regenerative medicine therapies due to their immunomodulatory capacity. In this context, the endocannabinoid system (ECS) has been identified as mediating the immune-stem cell dialogue, even if no information on AEC is available to date. Therefore, this study was designed to assess whether ECS is involved in tuning the constitutive and lipopolysaccharide (LPS)-induced ovine AEC anti-inflammatory and pro-inflammatory interleukin (IL-10, IL-4, and IL-12) profiles. Firstly, interleukins and ECS expressions were studied at different stages of gestation. Then, the role of cannabinoid receptors 1 and 2 (CB1 and CB2) on interleukin expression and release was investigated in middle stage AEC using selective agonists and antagonists. AEC displayed a degradative more than a synthetic endocannabinoid metabolism during the early and middle stages of gestation. At the middle stage, cannabinoid receptors mediated the balance between pro-inflammatory (IL-12) and anti-inflammatory (IL-4 and IL-10) interleukins. The activation of both receptors mediated an overall pro-inflammatory shift—CB1 reduced the anti-inflammatory and CB2 increased the pro-inflammatory interleukin release, particularly after LPS stimulation. Altogether, these data pave the way for the comprehension of AEC mechanisms tuning immune-modulation, crucial for the development of new AEC-based therapy protocols.

## 1. Introduction

The amnion (AM) is a fetal membrane that completely surrounds the developing fetus. It is responsible for fetus nutrition via diffusion and pregnancy homeostasis preservation [1,2,3]. The AM compartments (epithelial layer, basement membrane, and mesenchymal layer) generate a physical and immunological barrier that protects the fetus, triggering defense mechanisms against possible pathogens and maintaining the amniotic cavity sterile [4].

These peculiar biological properties have attracted the attention of researchers and clinicians that exploited AM anti-inflammatory, anti-fibroblastic, and anti-microbial properties for several tissue engineering applications [5,6,7,8,9,10].

Most recently, regenerative medicine protocols have included AM as a source of amniotic-derived stem cells. Interestingly, amniotic epithelial cells (AEC), which form the lining monolayer in direct contact with the amniotic fluid, have been largely used to this biomedical aim [11,12].

Increasing evidence has demonstrated that AEC display a great therapeutic potential, responding to the urgent need of a plentiful, safe, and ethically acceptable stem cell source for the development of regenerative medicine protocols to restore functionality in damaged or diseased tissues [13,14].

Of note, AEC conjugate a high plasticity, probably related to their early embryonic development [12,13,15], with a great ability to produce growth factors sustaining key processes involved in tissue regeneration such as restraint of fibrosis and immunosuppression via inhibition of both constitutive and lipopolysaccharide (LPS)-induced immune systems [2,14,16,17,18,19].

The high regenerative potential of AEC has been related, in particular, to their great ability in regulating inflammation [13]. Inflammation is a beneficial defensive response that can remove pathogens, repair injured tissues, and restore homeostasis to damaged tissues and organs. A prompt self-limiting inflammation is an essential early step for triggering a proper repairing process [20]. In an opposite manner, the persistence of a chronic status of inflammation can be responsible for tissue healing deregulation evolving in pathologies, including fibrosis and autoimmune diseases with subsequent loss of tissue functions. Moreover, chronic inflammation fuels tumor development as well as the ability for tumor cells to metastasize [21,22,23].

Amniotic-derived cells contribute to modulate these complex biological events through recognized paracrine mechanisms that physiologically modulate inflammation in the maternal–fetal interface and generate compensatory mechanisms maintaining this tolerance during pregnancy [24]. They include the secretion of cytokines and growth factors with antiapoptotic, proangiogenic, and immune-regulatory molecules [25,26,27,28,29,30,31] that overall contrast pro-inflammatory signals (cytokines and cells), thus enhancing anti-inflammatory, pro-regenerative immune pathways. Amongst them, it has recently been demonstrated in the amniotic epithelium that a highly conserved physiological mechanism controlling the expression and releasing balance of potent anti-inflammatory and pro-inflammatory cytokines such as interleukin (IL)-10, IL-4, and IL-12 under both basal and LPS-inductive conditions [24,32,33,34].

Although little is known about the effector role of these cytokines, amnion-mediated IL-10 modulation has been particularly ascribed to resolve the inflammatory process associated with intra-uterine infection-associated preterm labor [35].

In light of these findings, the exploitation of AEC’s anti-inflammatory and immunomodulatory properties such as the endocannabinoid system (ECS), as well as their control mechanisms, assumes a potential therapeutic impact, opening the need to improve knowledge on the molecular mechanism safeguarding cell homeostasis.

The ECS, a complex cell-signaling network of bioactive lipids regulating cell proliferation, differentiation, and survival has been recognized as a powerful modulator of inflammatory cell mediated-responses in mesenchymal stromal cells (MSC) and immune cells [36,37]. Their cytokine release was related to endocannabinoids (EC), such as anandamide (AEA) and 2-arachidonoylglycerol (2-AG), along with a set of receptors and enzymes that regulate their synthesis and degradation, acting as secondary modulators and increasing or decreasing a plethora of immune functions. In particular, EC bind to type-1 (CB1) and type-2 (CB2) cannabinoid receptors, as well as to the transient receptor potential vanilloid 1 (TRPV1) ion channel. Different enzymes are involved in AEA and 2-AG metabolism: an *N*-acylphosphatidyl ethanolamine (NAPE)-specific phospholipase D (NAPE-PLD) that synthesizes AEA, which is cleaved by fatty acid amide hydrolase (FAAH); 2-AG instead is mainly synthesized by two specific *sn*-1 diacylglycerol-lipases α and β (DAGL α/β), and is degraded by a specific monoacylglycerol lipase (MAGL) [38].

Over the last decade, several works have demonstrated the role of cannabinoid receptors (CB) in mediating immunomodulatory effects in immune cells. The CB2 was shown to modulate immune cell functions, both in cellular (leukocytes and T lymphocytes) and in animal models of inflammatory diseases [39,40].

The role of CB1 in immune functions has been deciphered using various approaches, including CB1-selective agonists and antagonists, and *CB1 ^−/−^* mice [41]. Gowran et al. described a key role for CB1 receptor type, propping up pro-survival functions during acute stress in a stem cell model [37].

Interestingly, the expression of the ECS was recently confirmed to control hematopoietic and neural stem cells’ immunomodulatory activities [42,43]. Rossi et al. not only demonstrated that human bone marrow-derived MSC (BM-MSC) express all ECS components, but they even clarified that the cell anti-inflammatory properties are enhanced by the activation of CB2 that, in turn, improves cell survival and capability to home and migrate towards endocannabinoid sources [42].

In addition, a central role of ECS in the regenerative tissue mechanisms seems to be confirmed by the cell-to-cell cross-talk demonstrated between BM-MSC and the inflammatory cell compartments [43].

Because no previous knowledge on the ECS role on AEC’s immunomodulatory properties is available to date, the present study was designed to investigate the expression profiles of the main ECS components (metabolic enzymes and receptors) and of the major key anti-inflammatory and pro-inflammatory interleukins (*IL-4*, *IL-10*, and *IL-12*, respectively) during the different stages of gestation using ovine AEC as stem cell source. The ovine model has been chosen on the basis of its high translational value, its great use in pre-clinical studies, and in order to have a large availability of cells at different gestational stages (early, middle, and late stage) without any ethical concerns [13].

Our data show how the interleukin expression and releasing profiles in AEC are both under the control of CB, thus paving the way to the comprehension of the mechanism regulating the cross-talk between fetus, uterus, and amnion in pregnancy, as well as supporting the development of innovative AEC endocannabinoid-based immunotherapeutic strategies for targeted regenerative medicine solutions.

## 2. Materials and Methods

### 2.1. Ethics Statement

No ethics statement is required for the present research. The amniotic membranes used in the research were collected from waste reproductive tissues of animals slaughtered for feed purposes.

### 2.2. Reagents

Ultra-pure grade chemicals were used: Progesterone (4 pregnene-3,20-dione, P_4_), lipopolysaccharide (LPS, from Escherichia coli 055:B5), CP55,940, and SR144528 (CB2 antagonist) were purchased from Sigma-Aldrich Corp. (St. Louis, MI, USA). ACEA (CB1 agonist) and AM281 (CB1 antagonist) were obtained from Tocris Bioscience (Bristol, United Kingdom), and JWH-015 (CB2 agonist) was obtained from Enzo Life Sciences (Plymouth Meeting, PA, USA). [^3^H]CP55,940 was obtained from Perkin-Elmer Life Sciences, Inc. (Boston, MA, USA).

### 2.3. AEC Isolation

AEC were obtained from uteri collected from sheep of Appeninica breed (2–3 years old and of approximately 50 kg) sacrificed at a local slaughterhouse. Fetus dimension was used to evaluate the stage of gestation. Early, middle, and late stage AM were collected from fetuses sized 8–10 cm (approximately first month of pregnancy), 20–25 cm (approximately 2.5 months), and 35–40 cm (more than 4 months gestation), respectively [44]. Once the uterus wall was opened, AM located in the opposite area to umbilical cord were isolated and mechanically peeled off from the chorion with the aid of a stereomicroscope. The avascular tissues were then reduced in pieces of 3–5 cm, washed in phosphate-buffered saline (PBS), and incubated in 0.25% trypsin/EDTA (EthylenDiaminoTetracetyc Acid) 200 mg/L at 37.5 °C for 20 min with continuous gentle shaking. The debris released during this digestion step were discarded. The cell suspension obtained after the enzymatic digestion was collected, filtered through a 40 µm cell filter (Sigma-Aldrich Corp., St. Louis, MI, USA), and collected into a tube containing 10% fetal calf serum, (FCS; Lonza, Basel, Switzerland) in order to inactivate trypsin. After centrifugation, viable cells were counted by means of a hemocytometer chamber following the trypan-blue staining. Freshly collected cells were used for the detection of ECS expression. On the contrary, AEC were incubated as specified below in order to perform the other analytic approaches.

### 2.4. Cell Culture and CB Agonist/Antagonist Treatments

Isolated AEC were seeded at 3 × 10^3^ cells/cm^2^ and cultured until 80% confluence in alpha Eagle’s minimum essential medium (α-MEM; Gibco; Thermo Fisher Scientific, Inc., Waltham, MA, USA) supplemented with 20% FCS, 25 μM P_4_ (Sigma-Aldrich Corp., St. Louis, MI, USA), 1% ultra glutamine (Lonza, Basel, Switzerland), 100 U/mL penicillin (Lonza Basel, Switzerland), 100 μg/mL streptomycin (Lonza Basel, Switzerland), and 2.5 μg/mL amphotericin (EuroClone Spa, Pero, MI, Italy), and incubated at 38.5 °C in 5% CO_2_ according to Canciello et al. [32]. This typology of cells was used in order to test CB binding. In addition, constitutive and LPS-induced interleukin expression profiles of AEC were compared by treating AEC monolayer without or with LPS (1 μg/mL) for 24 h. The modulatory effect of CB on the expression and releasing of interleukins was tested by exposing AEC to CB1 and CB2 agonists and antagonists. To this aim, AEC were exposed for 1 h to CB treatment in serum-free medium. Then AEC incubation continued for a further 23 h in cultural medium supplemented with 20% FCS. AEC were then collected for interleukin real-time qPCR analyses, whereas conditioned media (CM) were used for cytokines release detection. CB modulation was carried out using a CB1 agonist and antagonists ACEA (1 μM) and AM281 (1 μM) [45]. CB2 activation and inhibition [46,47] were induced by adding the selective agonist JWH-015 (1 μM) and the antagonist SR1445282 (1 μM).

### 2.5. Real-Time qPCR

Freshly collected AEC were used for the analysis of ECS expression profile. Briefly, total RNA was extracted with Trizol reagent (Sigma-Aldrich Corp., St. Louis, MI, USA) following the manufacturer’s instructions. Samples were then treated with DNase-1 (Sigma-Aldrich Corp., St. Louis, MI, USA), and 1 μg of total RNA was retrotranscribed using Random Hexamers (Bioline, London, UK) and Tetro Reverse Transcriptase (Bioline, London, UK), following the manufacturer’s instructions. PCRs were carried out in triplicate using the SensiFAST SYBR Lo-ROX kit (Bioline London, UK) on a 7500 Fast Real-Time PCR System (Life Technologies, Carlsbad, CA, USA), according to the manufacturer’s instructions. The following PCR conditions were used for all experiments: 95 °C for 10 min, followed by 40 cycles at 95 °C for 10 seconds and 60 °C for 30 s. Relative quantification was performed by using the ΔΔCt method. *GAPDH* (Glyceraldehyde 3-phosphate dehydrogenase) was selected amongst housekeeping genes for gene quantification. Primer sequences used in this manuscript are reported in Table 1.

### 2.6. Cannabinoid Receptor Binding Assay

Receptor binding assay was performed on viable AEC, incubated as described above with the CB1/CB2 radiolabelled agonist [^3^H]CP55,940 (Perkin-Elmer Life Sciences, Boston, MA, USA) at different concentrations (0.5, 1, and 2.5 nM) in the presence of 50 mM Tris-HCl, 1 mM CaCl_2_, 5 mM MgCl_2_, and 0.2% BSA (Bovine Serum Albumin), pH 7.4 at 37 °C for 1 h. After this, the incubation buffer was removed and the cells were washed twice with 1 mL of ice-cold washing solution (50 mM Tris-HCl with 1% BSA). Subsequently, 500 μL of 0.1 M NaOH was added to each sample, and the cells were collected and transferred into a scintillation tube to test radioactivity. In all binding experiments, nonspecific binding was determined in the presence of 1 μM “cold” agonist CP55,940.

### 2.7. ELISA Detection of Interleukins in AEC CM

IL-4, IL-10, and IL-12 levels in CM collected from AEC cultures in the presence or absence of CB agonist and antagonists were measured using Nori Sheep IL-4, IL-10, and IL-12 ELISA kits (Genorise Scientific, Inc.; Glen Mills, PA, USA). CM were added to the microtiter plate wells with a horseradish peroxidase (HRP)-conjugated antibody and were processed according to manufacturer’s instructions. The optical density of each well was determined by using a microplate reader set to 450 nm and subtracting the corresponding reading at 540 nm for each well.

### 2.8. Statistical Analyses

Data reported are the mean (± SD) of at least three independent experiments, each performed in triplicate. All statistics were performed using Prism 6 program (GraphPad Software for Science, San Diego, CA, USA). Statistical analysis was performed using Student’s *t*-tail test. Probability of at least *p* < 0.05 was considered statistically significant.

## 3. Results

### 3.1. The ECS Was Modulated in AEC during Pregnancy

**Figure 1:**
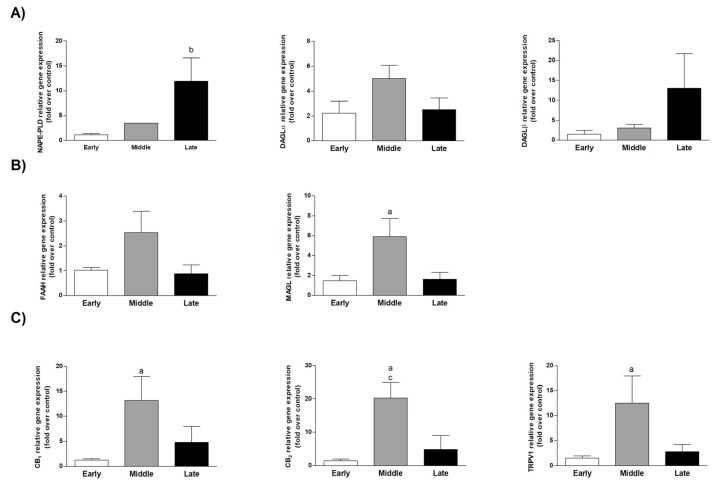
Endocannabinoid system (ECS) gene expression modulation in amniotic epithelial cells (AEC) collected at different stages of gestation. Real-time qPCR gene expression analyses of enzymes involved in the anandamide (AEA) and 2-arachidonoylglycerol (2-AG). (**A**) Synthesis (*N*-acylphosphatidyl ethanolamine (NAPE)-specific phospholipase D (NAPE-PLD), diacylglycerol-lipases α and β (DAGLα/β), (**B**) degradation (fatty acid amide hydrolase, FAAH), monoacylglycerol lipase (MAGL)), and (**C**) receptors (cannabinoid receptor (CB)1, CB2, transient receptor potential vanilloid 1 (TRPV1)). Each analysis was performed using AEC isolated at different stages of fetal development. Relative quantification was performed using the ΔΔCt method. *GAPDH* was used as housekeeping gene quantification. Data are the mean ± SD obtained from at least *n* =  3 independent experiments performed using two different fetuses (^a^ middle vs. early; ^b^ late vs. early; ^c^ middle vs. late).

The expression of ECS key genes such as metabolizing enzymes (*NAPE-PLD*, *FAAH*, *DAGLα/β*, and *MAGL*) and CB (*CB1*, *CB2*, and *TRPV1*) was assessed by using cells freshly isolated from AM epithelial layer collected at early (first month), middle (2–2.5 months), and late (3.5–4 months) stages of gestation [32]. The results showed that ECS was steady expressed in AEC, even if differently modulated during gestation (Figure 1).

Apart from *NAPE-PLD*, whose expression prevailed in cells isolated at late stage of gestation (*p* < 0.05 late vs. early stage cells), *MAGL* (*p* < 0.05 middle vs. early stage cells; Figure 1B), both extracellular *CB1* and *CB2* (*p* < 0.05 middle vs. early, *p* < 0.01 middle vs. early, and *p* < 0.05 middle vs. late stage cells; Figure 1C), and the intracellular receptors *TRPV1* (*p* < 0.05 middle vs. early stage cells; Figure 1C) were all up-regulated in the middle stage AEC.

### 3.2. Cannabinoid Receptor Binding Activity of AEC Was Higher at Middle and Late Stage of Gestation

The study of the CB signaling in AEC was undertaken, firstly by testing the ability of the synthetic radio-labelled CB1/CB2 receptor agonist CP55,940 to bind to CB1 and CB2 receptors in AEC collected at different gestational stages.

**Figure 2:**
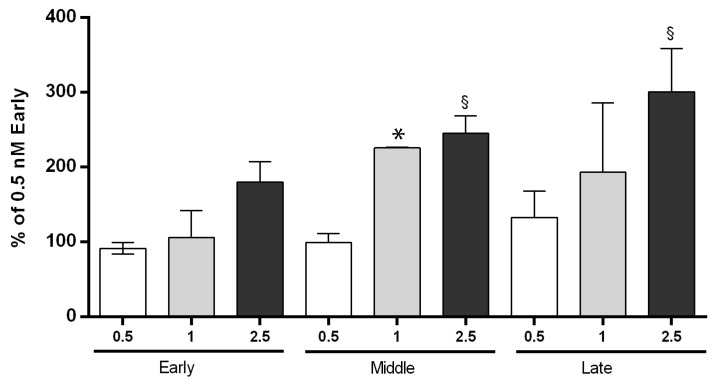
CB binding affinity in AEC in early, middle, and late stages of gestation. The binding activity assay was performed on intact cells at different CP55,940 concentrations (0.5, 1, and 2.5 nM) and gestational stages (early, middle, and late). Data are the mean ± SD obtained from *n* = 3 independent experiments performed using three fetuses for each gestational stage (in each gestational stage * 1 vs. 0.5; ^§^ 2.5 vs. 0.5).

The higher CP55,940 binding activity was observed in the middle and late phase of AEC. On the basis of observed data at 1 and 2.5 nM dose points (Figure 2), it appeared evident that binding activity was dose-dependent.

In more detail, a significant modulation of CB was remarkable at doses greater than 1 nM. At lower doses, the amount of radioligand recruited only a small fraction of the total binding sites. In addition, an enhanced activity was recorded in AEC isolated at the middle stage of gestation. These cells displayed, indeed, significantly higher binding activities either at 1 and 2.5 nM of CP55,940 (Figure 2), whereas a significant modulation of CB was evident in late cells exclusively at the greatest radio-ligand concentration (2.5 nM: Figure 2). These data unveiled a considerable regulation of CB binding activity in AEC, with a significant effect during the transition from early to middle/late stage of gestation.

### 3.3. Interleukin Expression Profile Was Strictly LPS- and Gestational-Dependent

**Figure 3:**
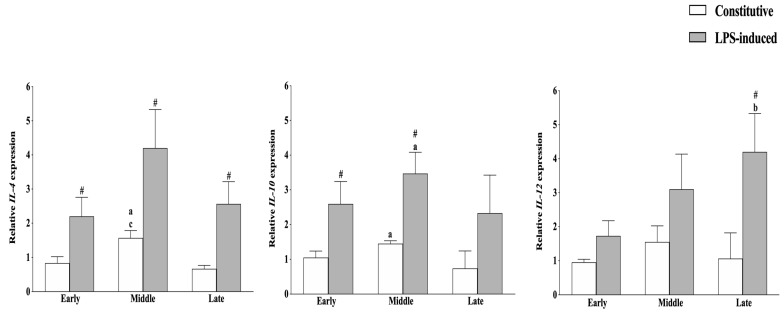
Anti-inflammatory and pro-inflammatory interleukin expression profile modulation during gestation in AEC. Constitutive (white bars) and lipopolysaccharide (LPS)-induced (grey bars) interleukin (*IL*)*-10*, *IL-4*, and *IL-12* mRNA expressions were studied in AEC isolated at different stages of gestation. Relative quantification was performed using the ΔΔCt method. *GAPDH* was used as housekeeping gene quantification. The data are mean ± SD of three replicates (*n* = 3 experimental replicates) performed in AEC collected from at least two fetuses/gestation stages (*n* = 2 biological replicates). Statistical analysis was performed using Student’s *t*-tail test. *p* < 0.05 was considered statistically significant. (**^#^** LPS vs. constitutive (*CTR*); ^a^ middle vs. early; ^b^ late vs. early; ^c^ middle vs. late).

To get insight into immunomodulatory properties of AEC during gestation, firstly, the gene expression analysis of key anti-inflammatory (*IL-4* and *IL-10*) and pro-inflammatory (*IL-12*) interleukins in the constitutive and LPS-activated conditions were performed without taking into account the CB modulatory effect. As reported in Figure 3, the constitutive interleukin expression profile was only slightly affected by the gestation stage, with the exception of the *IL-4* gene, whose transcription was significantly higher in cells collected at the middle phase (*p* < 0.05 constitutive expression in middle vs. late and vs. early).

LPS treatment induced an overall up-regulation of the anti-inflammatory interleukins at early and middle stages of pregnancy. More in detail, LPS significantly increased *IL-10* expression in these typologies of cells while up-regulating *IL-4* independently of AEC gestational stage (Figure 3).

Conversely, LPS treatment induced a pro-inflammatory profile at the late stage of pregnancy by inducing *IL-12* up-regulation (*p* < 0.05 LPS-induced vs. constitutive *IL-12*; Figure 3) in AEC. In more detail, *IL-12* mRNA reached levels significantly higher than those recorded in early isolated cells (*p* < 0.01 LPS-induced *IL-12* expression in late vs. early cells).

### 3.4. ECS Controlled the Immune Activities of AEC at Middle Stage of Gestation

In order to evaluate the influence of extracellular CB modulation on constitutive (CTR) and LPS-induced (LPS) interleukin expression and release, AEC derived from the middle stage of gestation were exposed to CB1 and CB2 selective agonists (ACEA and JWH-015, 1 μM) or antagonists (AM281 and SR1445282, 1 μM).

Given the widespread use in regenerative medicine of preclinical-related studies of the AEC isolated in the middle phase of pregnancy [13], their great availability, and their major ECS sensitivity as demonstrated in previous experiments, AEC exclusively isolated from AM at the middle stage of gestation were used for studying the ECS role on immunomodulatory activity.

#### Interleukin Gene Expression Profile in AEC Was Mainly Modulated by CB2

In the constitutive condition, AEC exposed to selective CB2 agonist JWH-015 displayed a significant *IL-12* up regulation. The specificity of CB2 role was also confirmed through the use of the CB2 antagonist SR1445282, which reversed *IL-12* expression to constitutive levels.

**Figure 4:**
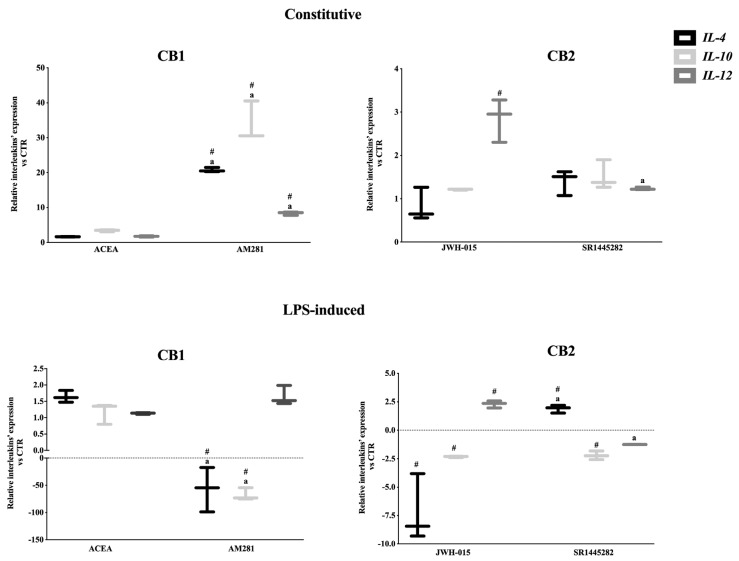
CB controlled both constitutive and LPS-activated interleukin expression in the middle stage of gestation. Comparison between mRNA profiles of key anti-inflammatory (*IL-4* and *IL-10*) and pro-inflammatory interleukin (*IL-12*) recorded in middle stage AEC after 24 h of culture in the absence (constitutive) or presence of LPS stimulus (LPS-induced). Gene profiles were assessed by exposing the cells to 1 μM of selective CB1 or CB2 agonists (ACEA or JWH-015, respectively) or antagonists (AM281 or SR144282, respectively). Relative quantification was performed using the ΔΔCt method. *GAPDH* was used as housekeeping gene quantification. The data are mean ± SD of three independent replicates (*n* = 3 experimental replicates) performed using two different fetuses at middle stage of gestation (*n* = 2 biological replicates). Statistical analysis was performed using Student’s *t*-tail test. *p* < 0.05 was considered statistically significant (^#^ values significantly different from CTR; ^a^ value statistically different between agonist and antagonist treatments).

Conversely, CB2 activation did not affect the expression of the anti-inflammatory interleukins (Figure 4).

On the other hand, interleukin expression levels were not influenced by the CB1 activation, as demonstrated by using the selective agonist ACEA. Instead, AEC exposure to the CB1 selective antagonist (AM281) promoted an overall immune activation.

Of note, the LPS-mediated interleukin expressions were analogously influenced by CB2 (Figure 4). Indeed, JWH-015 promoted the down-regulation of both anti-inflammatory interleukins and, at the same time, the up-regulation of IL-12. In more detail, CB2 stimulated an intense pro-inflammatory response in AEC exposed to LPS by lowering ≅7 and ≅2 times the expression of IL-4 and IL-10, respectively, and by doubling the mRNA levels of IL-12 (Figure 4). Of note, SR1445282 treatment reverted the pro-inflammatory expression pattern with the unique exception of IL-10, which remained down-regulated (Figure 4). CB1 activation did not affect interleukin expression, whereas its antagonist triggered a dramatic down-regulation of both anti-inflammatory interleukins.

In summary, CB2 activation triggered pro-inflammatory feedback in middle stage AEC. However, the CB2-mediated effect operated through a constitutive overexpression of IL-12, whereas it induced IL-12 up-regulation and a combined IL-4 and IL-10 inhibitory response after LPS stimulation.

### 3.5. Interleukin Release Was CB-Dependent

**Figure 5:**
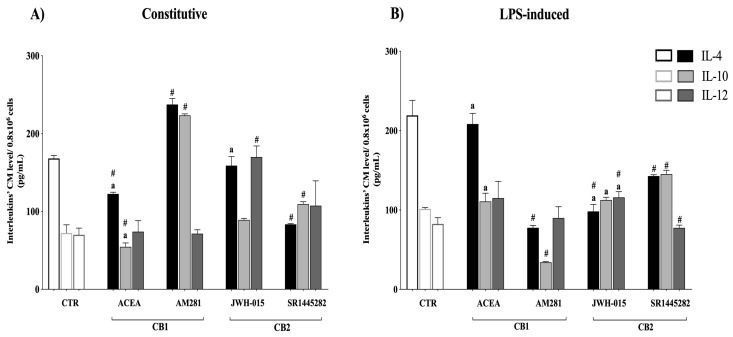
Constitutive and LPS-induced interleukin releasing response in the middle stage of gestation was under CB1 and CB2 modulation. Comparison between protein profiles of anti-inflammatory (IL-4 and IL-10) and pro-inflammatory cytokines (IL-12) measured in conditioned media (CM), collected from the middle stage of AEC incubated for 24 h in absence (**A**) constitutive release) or in the presence of LPS stimulus (**B**) LPS-induced release). Protein profiles were ELISA-assessed by exposing the cells to the selective CB1 or CB2 agonist (ACEA or JWH-015, respectively; 1 μM) and antagonist (AM281 or SR144282, respectively; 1 μM). The data reported are the mean ± SD obtained from at least *n* = 3 independent experiments (*n* = 3 experimental replicates) performed using two different fetuses at middle stage gestation (*n* = 2 biological replicates). Statistical analysis was performed using Student’s *t*-tail test. *p* < 0.05 was considered statistically significant (^#^ indicates values significantly different from CTR; ^a^ indicates values significantly different between agonist and antagonist treatments).

In order to obtain further insights into the mechanisms underlying the CB-mediated immunomodulatory activity in AEC, the interleukin release was measured by an ELISA assay. The results showed that the activation of both CB modulated the constitutive interleukin by releasing activities in AEC. In more detail, CB1 activation significantly decreased IL-4 and IL-10 release (both *p* < 0.05 ACEA vs. CTR). Importantly, the specificity of CB1 action was confirmed by the opposite releasing response induced by exposing AEC to CB1 antagonist (Figure 5).

CB2 activation promoted an IL-12-dependent pro-inflammatory response. Indeed, AEC exposed to the CB2 selective agonist significantly increased IL-12 levels (*p* < 0.01 JWH-015 vs. CTR). The specificity of such CB2 activation was confirmed by the ability of CB2 antagonist to reduce IL-12 levels back to pretreatment values.

Conversely, the LPS-activated interleukin releasing profile of AEC was exclusively under CB2 control. Indeed, AEC exposed to CB2 selective agonist activated a combined pro-inflammatory response involving a dramatic IL-4 decline and a significant IL-12 increase (both *p* < 0.01 JWH-015 vs. CTR levels) (Figure 5). CB2 antagonist treatment caused an opposite effect—IL-4 and IL-10 increased, reaching levels higher than those induced by JWH-015 (both *p* < 0.05), whereas IL-12 dropped (*p* < 0.05 vs. JWH-015). On the contrary, CB1 activation did not affect interleukin releasing in the LPS-induced state (Figure 5).

Overall, ECS activation triggered an overall pro-inflammatory response in AEC by reducing anti-inflammatory (IL-4 and 10) and increasing pro-inflammatory (IL-12) interleukin releasing upon CB1 and CB2 activation, respectively.

### 3.6. AEC’s Immunomodulatory Activity Was Dependent on CB Receptor Signaling

**Figure 6:**
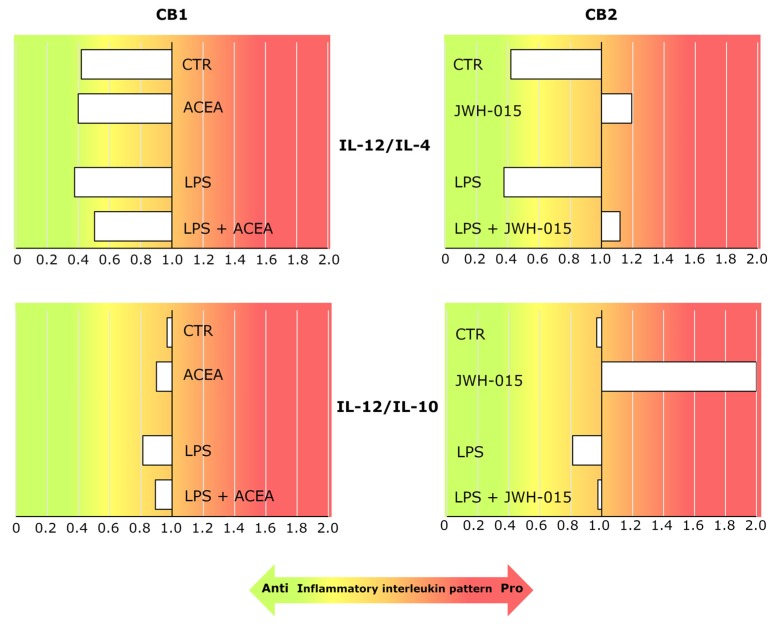
CB2 activation modulated the immune activity of AEC in both constitutive and LPS-induced conditions by triggering a pro-inflammatory releasing profile of interleukins in the middle stage of gestation. The histograms show the ratios between IL-12 and both the anti-inflammatory cytokine (IL-4 or IL-10) levels in the CM under constitutive and LPS-induced conditions, in the presence or absence of 1 μM CB1 or CB2 agonists (ACEA or JWH-015, respectively). The background color gradient indicates the AEC immune activity pattern—red and green colors for the pro-inflammatory (ratios > 1) and anti-inflammatory (ratios < 1) profiles, respectively.

Finally, the interleukin release in AEC was expressed by using the ratio between the pro-inflammatory (IL-12) and both anti-inflammatory (IL-4 and IL-10) interleukin levels measured in the CM (Figure 6), in order to summarize the overall immunomodulatory effect mediated by CB under both constitutive and LPS-activated conditions.

The ratios support a model where AEC predominantly expressed an anti-inflammatory interleukin releasing profile in both constitutive (ratios < 1 of both IL-12/IL-4 and IL-12/IL-10 under CTR) and LPS-induced (ratios < 1 of both IL-12/IL-4 and IL-12/IL-10 under LPS conditions) conditions.

CB1 activation only slightly affected this AEC anti-inflammatory profile (ratios < 1 of ACEA induced IL-12/IL-4 and IL-12/IL-10 both under CTR and LPS conditions).

On the contrary, CB2 stimulation mediated an overall pro-inflammatory profile in both constitutive and LPS-induced conditions, as confirmed by the IL-12/IL-4 (both JWH-015 and LPS + JWH-015 ratios > 1) and the IL-12/IL-10 (both JWH-015 and LPS + JWH-015 ratios ≥ 1) ratio reversing. Of note, the pro-inflammatory resect of AEC was particularly evident under interleukin constitutive releasing conditions.

## 4. Discussion

The amnion has a key role in the establishment, progression, and successful outcome of pregnancy. Specifically, the amniotic innermost epithelial compartment of the amnion creates an immunological barrier protecting the fetus. Due to its early gestational stage of development [48] and embryonic origin (epiblast), the amniotic innermost epithelial compartment offers a large source of AEC that combines great stemness properties with constitutive immunomodulatory attitudes [13,49].

**Figure 7:**
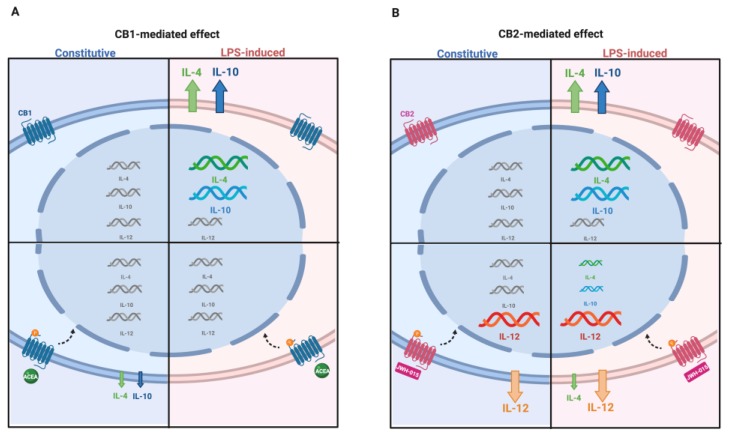
ECS-dependent interleukin release in AEC under constitutive and LPS-induced conditions. The picture summarizes the modulatory effect of ECS on pro- and anti-inflammatory interleukin expression and releasing profiles in the middle stage of gestation. The modulation of interleukin gene expression is indicated by means of different acid nucleic sizes. Changes in quantity of released/secreted interleukin proteins is indicated by different arrow sizes (upregulation and downregulation). (**A**) CB1 activation by agonist ACEA; (**B**) CB2 activation by agonist JWH-015 in the constitutive (blue panel) and LPS-induced (pink panel) conditions.

The present research represents the first piece of evidence indicating that AEC express and modulate ECS during pregnancy. Interestingly, a CB-mediated effector response was observed in AEC collected at the middle stage of gestation (Figure 7), aiming to balance pro-inflammatory (IL-12) and anti-inflammatory (IL-4 and 10) interleukin expression and release.

These findings improved the knowledge on the AM cell mechanism and, at the same time, open new frontiers in exploiting AEC immunosuppressive activities for cell-based therapy purposes.

In more detail, AEC expressed a fully functional ECS characterized by a gestational stage-dependent modulation of both metabolic enzymes (NAPE-PLD, FAAH, DAGL, and MAGL) and CB (CB1, CB2, and TRPV1).

On the basis of the present results, the AEC showed a very low expression profile of ECS genes at the early stage of gestation. During pregnancy, ECS sensitivity increased through the up-regulation of receptor expression (CB1-CB2 and TPRV1) and binding activity of both extracellular forms. At the same time, at middle/late stage of gestation, the AEC could affect the local endocannabinoid tone by modulating enzymes, controlling the levels of 2-AG and AEA, the most biologically active EC. Accordingly, the up-regulation of the degradation 2-AG enzyme (MAGL) characterized the middle stage. Furthermore, in the late gestational stage, AEC modified their ECS profile by acquiring a pro-active role in the synthesis of AEA with a significant NAPE-PLD overexpression. This latter ECS asset may contribute to the major concentration of EC in late pregnancy, according to the literature data where ECS has been reported as a key regulator of the final event of pregnancy [50]. In particular, human placenta is a likely target for EC action and metabolism, and consistently CB1 and FAAH are detectable in amniotic epithelial cells, chorionic cytotrophoblast, and especially in syncytiotrophoblast [51]. Of note, spatiotemporal distribution of AEA undergoes specific changes in order to support pregnancy onset; during the early stage at the implantation site, low levels are needed to promote uterine receptivity and maintenance of pregnancy, whereas high levels support parturition at the time of labor, probably because AEA is hydrolyzed and releases arachidonic acid, in turn increasing the concentration of prostaglandins [50,51].

The present study enlarged the evidence collected to date supporting the role for ECS as a physiological modulator of reproduction [51]. As previously demonstrated, during the early stage post-fertilization, low levels of AEA and 2-AG are required to support implantation [52,53,54].

An aberrant expression of AEA metabolic enzymes and CB are associated with a compromised trophoblast differentiation occurring in deficit of implantation [52,55], with a greater risk of miscarriage in early pregnancy [56,57]. Accordingly, the epithelial layer of the amnion characterizing the early and middle stages might contribute to this permissive reproductive framework by maintaining a prevalent degradative ECS expression pattern that might contribute to keep low levels of EC required for successful implantation and pregnancy progression. On the contrary, high levels of EC will be then required at labor in order to stimulate amnion to produce prostaglandins via CB1 [58]. AEC may also affect this ECS asset by switching on NAPE-PLD, thus reinforcing the local levels of bioactive lipids. This additional evidence seems to reinforce the hypothesis that AEC exert a local contribution in triggering labor [57,59].

The modulatory role of AEC during pregnancy may be also due to their cytokine releasing profile, representing one of the most relevant maternal–fetal signaling pathways balancing the pregnancy maintenance and labor triggering mechanism. Local pro-inflammatory cytokine and chemokines have been implicated in the pathophysiology of human labor since the 1980s. More recently, robust evidence has linked their intrauterine production increase to both term and preterm labor [60]. Taking into account the present results, it appears evident that AEC are able to make a fine balance between locally produced pro-inflammatory and anti-inflammatory cytokines, which is critical for the success of pregnancy [61], by using two different approaches. First of all, AEC are able to maintain the local concentration of anti-inflammatory and pro-inflammatory interleukins under a steady control, taking advantage of their constitutive expression and release. However, it appears clear that in the presence of acute inflammatory stimuli, the AEC even reinforce their anti-inflammatory activity during the early and middle gestational stages. This seems to be a conservative mechanism that is able to increase the expression of anti-inflammatory cytokines in response to LPS stimulation, thus generating a compensatory mechanism that may limit the secretion of pro-inflammatory modulators and that could jeopardize the immune privilege during pregnancy by triggering pre-term labor [24,32].

However, this behavior seems to be active exclusively at the beginning of gestation, whereas at term, any inflammatory mimicking event induces a dramatic up-regulation of pro-inflammatory inflammatory cytokine such as IL-12. This is in agreement with the evidence showing an increased amount of inflammatory cytokines [62,63] that are known to increase in amniotic fluid towards term, at least in humans. This phenomenon plays a role in labor by stimulating a local production of prostaglandins (PGs) and collagenase [64]. Pregnancy mechanisms have often been compared with those involved in organ allotransplantation because both the fetus and placenta have to be considered as semi-allografts structures expressing both maternal and paternal antigens [65]. Labor, like rejection, has been frequently related to the breakdown of fetal-maternal immune tolerance, with variable consequences depending on the gestation: recurrent miscarriages [66], pre-term labor [67], and pre-eclampsia [68]. Tolerance is maintained via factors produced at the implantation site, and also by an active role of amnion. One key molecule mediating tolerance is IL-10, an anti-inflammatory cytokine [69]. IL-10 was demonstrated to be a modulator of uterine natural killer (NK) cell cytotoxicity; in an IL-10-depleted mice model, very low doses of LPS led to uterine NK (uNK) cell activation and fetal demise [70]. In a non-human primate model, IL-10 was shown to inhibit IL-1β-induced uterine activity [71], and it seemed to also have an inhibitory effect on LPS induction of matrix metalloproteinase 2 (MMP-2) and 9 (MMP-9) in fetal membranes [72].

AEC may exert a positive role in the cell-to-cell cross-talk between fetus and inflammatory system involved in pregnancy progression by supporting the high IL-10 levels, either under constitutive or LPS-activated conditions [24,32], until the late stage when AEC becomes permissive to the interleukin framework, inducing labor.

However, this can only be considered a speculation until the releasing IL-10 profile is performed in AEC collected at late gestational stage.

Besides the physiological ECS role in pregnancy control, the impact that such a mechanism may have in modulating the constitutive and LPS-induced immune profile of AEC used in regenerative medicine protocols is equally remarkable.

Increasing evidence has demonstrated that AM-derived cells and their CM actively participate in the resolution of inflammation by acting through different immunosuppressive mediators. In the present research, it has been demonstrated that AEC displayed an overall immunosuppressive interleukin release that is operating either under constitutive or LPS-induced conditions (Figure 7). This peculiar modality of AEC to tune immunosuppression even in the absence of inflammatory conditions [27,49] may have a relevant impact because it can also be exploited in the absence of a large inflammatory stimuli such as under chronic disease processes. On the other hand, the immunosuppressive influence is potentiated under acute inflammatory conditions, as is the case in the presence of foreign pathogens or mechanical insults.

The results establishing a role for the ECS in both basal and LPS-stimulatory properties [24,32] hold the basis to improve the comprehension of the mechanisms underlining the duality of the immune response. This is an essential phase for the development of new endocannabinoid-based immunotherapeutic approaches to reach clinical benefit through the targeted modulation of the immune system.

Of note, these may be considered of value by considering the high translational value of ovine model either from a reproductive point of view (mono-ovulatory medium-sized mammal species) and for its great use, as demonstrated in preclinical models in AEC-based regenerative medicine studies [13].

In this context, even if CB1 did not appear to have any physiological role in controlling interleukin expression and release, the exacerbation of constitutive AEC immunosuppressive activities induced by its pharmacological inhibition opens new biotechnological solutions to potentiate cell- and cell-free-based AEC protocols that have been successfully proposed for the treatment of inflammatory disorders [7,73] such as liver, lung, and tendon fibrosis [31,74,75,76,77,78,79,80,81,82,83,84]; experimental autoimmune encephalo-myelitis [85]; traumatic brain injury [86,87]; and cardiac ischemia [88,89,90].

In addition, a balance between immunosuppression and immunostimulation may be carefully addressed for AEC before their clinical use. On the basis of the present results, it appears evident for the first time that the CB2 may be activated to revert the interleukin-mediated immune activity of AEC by transforming this stem cell source profile from immunosuppressive to immune-inductive.

Interestingly, the effects of CB1 and CB2 were demonstrated on the releasing protein profile of interleukin in order to rule out an over-estimation of their expression. This is all the more the case because the present data showed that the CB modulation sometimes differently affected the interleukin expression and releasing profile, thus highlighting the existence of mechanisms involved in post-translation.

CB2 activation reverted both the constitutive and LPS-induced releasing interleukin immune profiles by triggering pro-inflammatory activity in the middle stage of gestation (Figure 7). The CB2-mediated pro-inflammatory response of AEC was particularly effective under interleukin-constitutive releasing conditions, thus suggesting that such a condition may be induced exclusively by modulating the receptor-binding activity through biotechnological approaches. However, the use of stem cells to induce immunostimulation should not be excluded. Indeed, in multiple diseases characterized by an exacerbation of inflammatory conditions, the major need is to dampen the inflammatory process, but in other diseases, such as cancer, the stimulation of immune system has been proposed as an efficient therapeutic strategy [91].

## Figures and Tables

**Table 1:** Primer sequences used for real-time qPCR.

Sheep Gene	Forward (5′→3′)	Reverse (5′→3′)
*NAPE-PLD*	CTGTCTTGGGGCCTTGGAAC	GGCTCTAAATAATGCTCACTTGC
*FAAH*	CCTTGGGAGCAGAGGTTTCA	AGAGACTTGAGGTTGCTGGC
*DAGLα*	TGCTGAGCGAGGATGCTATG	CAAGTCACTGGGGTGAGTCC
*DAGLβ*	CACCGAGGTAGTCACCCACT	CTGATCACCTCCGACCAAGT
*MAGL*	GAAGCGACGTTCACAGGAGA	GCAGCAGTCCTGGAAGATCC
*CB1*	TGACCATGTCTGTGTCCACG	AGACGCTTCTGGGTTTCGAG
*CB2*	CATCGACCGCTACCTCTGTC	AGGTAGGACACCAATGCAGC
*TRPV1*	AACCAAGCCCCACAGCTTC	GGACAGCTGCCTGACACAC
*IL-4*	AAGCCCTCAGCTAAGCTCAAGTC	AGGCATCACAGGCTCAAGTC
*IL-10*	CCAGGATGGTGACTCGACTAG	TGGCTCTGCTCTCCCAGAAC
*IL-12*	TCAAACCAGACCCACCCAAG	CACAGATGCCCATTCACTCC
*GAPDH*	TCGGAGTGAACGGATTTGGC	CCGTTCTCTGCCTTGACTGT
